# Interface stability of intramedullary nail locking configurations under combined axial and torsional loading

**DOI:** 10.1007/s00068-026-03235-z

**Published:** 2026-07-08

**Authors:** Hollensteiner Marianne, Winkler Martin, Gerber Claus, Borowsky Anja, Augat Peter

**Affiliations:** 1 Institute for Biomechanics, BG Unfallklinik Murnau gGmbH, Prof. Küntscher Str. 8, 82418 Murnau, Germany; 2 Institute for Biomechanics, Paracelsus Medical University Salzburg, Salzburg, Austria; 3 Institute for Production Engineering and Machine Tools, Faculty of Mechanical Engineering, Leibniz University Hannover, Hannover, Germany; 4 Stryker Trauma GmbH, Schönkirchen/Kiel, Germany

**Keywords:** Intramedullary nailing, Locking configuration, Angle-stable fixation, Screw-bone interface, Synthetic bone model

## Abstract

The biomechanical performance of intramedullary nails in metaphyseal regions strongly depends on distal locking configuration. This study aimed to evaluate screw–bone interface stability under combined axial and torsional cyclic loading for different distal locking configurations of intramedullary nails, focusing on the effects of screw number, spatial distribution, interscrew distance, and multiplanar versus coplanar fixation using a standardized synthetic bone model. Six distal locking configurations (n = 5 per group) were tested under stepwise cyclic axial compression, superimposed with a constant torsional moment (± 2 Nm, 2 Hz). Primary endpoints were cycles to failure, cycles to 1 mm axial displacement, and cycles to 0.5° rotation. All constructs failed by progressive screw cut-through within the synthetic bone. The three-screw multiplanar configuration exhibited the highest endurance (≈ 36,000 cycles to failure) and lowest micromotion (≈ 34,000 cycles to 1 mm displacement), significantly outperforming the two-screw configurations overall (p = 0.002). Increasing interscrew distance from 10 mm to 34 mm improved migration resistance by approximately 25%, whereas the short 10 mm spacing led to premature failure. Blocked and free-hand configurations showed comparable behavior to the standard setup, indicating no added benefit from toggle elimination alone. Locking configuration decisively influences the performance of the implant-bone interface in intramedullary nail constructs. Multiplanar distal locking with adequate screw spacing enhances interface stability and may provide biomechanical advantages for metaphyseal fixation to reduce screw migration and early loss of fixation.

## Introduction

Intramedullary nailing represents the standard of care for the stabilization of long bone fractures due to preservation of fracture biology and favorable load-sharing characteristics [1–3]. The technique provides central alignment, allows for early weight bearing, and has demonstrated excellent clinical outcomes in diaphyseal fracture fixation [4]. As compared to plate fixation, and when both techniques are indicated based on fracture morphology, intramedullary nailing for selected metaphyseal fractures provides a central load-sharing construct that facilitates early weight bearing while requiring less soft-tissue dissection, which has been associated with lower rates of wound complications and infection. However, challenges remain when fractures extend toward the metaphyseal regions. In these cases, short cortical fragments, a limited number of possible screw holes, and reduced cortical thickness compromise the stability of nail anchorage [5]. Particularly in osteoporotic bone, loss of fixation and secondary fragment displacement continue to pose clinical problems, highlighting the need for optimized locking strategies that ensure durable fixation under physiological loading conditions [6, 7].

Over the past two decades, angular stability has become a key principle in osteosynthesis, as fixed-angle screw–implant connections reduce screw toggle and improve resistance to cyclic loading, particularly in locking plate systems [8, 9]. Similar concepts have been transferred to intramedullary nailing through angular-stable or “locking-in-locking” mechanisms [10–12], with biomechanical studies demonstrating increased stiffness and reduced interfragmentary motion compared to conventional free-motion locking. However, growing evidence suggests that overall construct performance is not determined by angular stability alone but is strongly influenced by screw-related parameters, including screw number, orientation, and spatial distribution within the bone segment [13–20]. In a systematic biomechanical investigation using a generic intramedullary nail–bone model, Hoffmann et al. demonstrated that angular-stable locking effectively reduces screw–nail clearance but has only minor and inconsistent effects on construct stiffness, whereas screw number and interscrew distance were the dominant determinants of mechanical performance under axial, torsional, and bending loading [21]. However, that study was limited to quasi-static testing and focused on stiffness and clearance measurements, without addressing cyclic fatigue behavior, progressive screw–bone interface damage, or failure mechanisms under combined physiological loading conditions.

A critical determinant of overall construct performance is the interface between the locking screws and the surrounding bone. Micromotions at this interface are inevitable during cyclic physiological loading and can lead to gradual cut-through of the screws, local bone resorption, and eventual loss of fixation [22]. These effects are particularly pronounced in low-density bone, particularly in constructs with reduced inter-screw spacing, thereby promoting stress concentration and premature fatigue failure [23]. Despite its biomechanical importance, the screw–bone interface in intramedullary nailing systems has been insufficiently characterized under combined loading conditions. Most available biomechanical studies on intramedullary nail locking have focused on global construct stiffness or failure under isolated axial or torsional loading conditions [11, 14–16]. While these approaches provide valuable insights into overall construct behavior, they do not adequately capture the cyclic, multiaxial loading environment encountered in vivo. As a result, important questions remain regarding the development of screw–bone interface micromotion, the onset of progressive screw cut-through, and the influence of screw number and spatial configuration under combined axial and torsional cyclic loading.

The present study aimed to systematically investigate the influence of specific distal locking configurations on screw–bone interface stability under combined axial and torsional cyclic loading. In particular, the effects of screw number (two versus three screws), screw orientation (medial–lateral versus anterior–posterior), interscrew distance (short versus wide spacing), and blocked versus non-blocked locking were analyzed using a standardized synthetic bone model. This approach allowed elimination of biological variability and enabled reproducible quantification of axial displacement and rotational micromotion at the nail–bone interface. We hypothesized that both screw number and spatial configuration significantly affect migration resistance and interface micromotion, with wider screw spacing and multiplanar fixation providing superior mechanical stability.

## Materials and methods

### Nail model

**Fig. 1:**
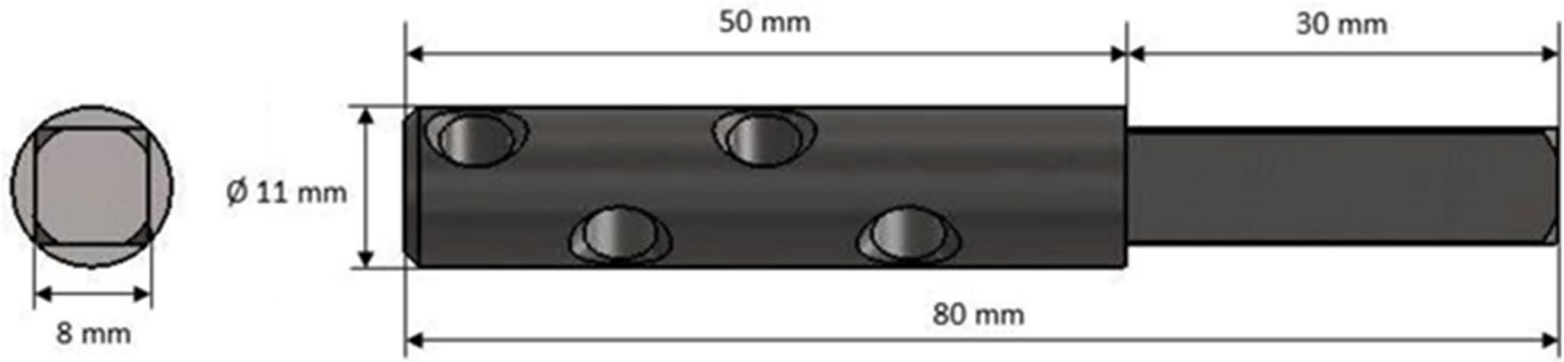
Dimensions of representative nail model with square cross-section for machine fixation (right) and four locking holes (left)

A generic intramedullary nail model was used, machined from solid titanium alloy (diameter 11 mm, length 80 mm) with a 30 mm proximal square section for fixation in the testing machine. Four drill holes for the distal locking screws (5.1 mm) were incorporated according to six defined locking configurations. All nails were manufactured by Stryker Trauma GmbH (Schönkirchen, Germany; Fig. 1).

### Bone model

The bone substitute consisted of rigid polyurethane foam cylinders (15 PCF, Sawbones Europe AB, Sweden) with a diameter of 27 mm and length of 90 mm, featuring a 17 mm central canal for nail insertion. The specimens were embedded in polymethyl methacrylate (PMMA; RenCast FC 53 A/B, Huntsman Advanced Materials, Basel, Switzerland) within aluminum pots to ensure rigid fixation in the testing machine.

### Specimen preparation

Six locking configurations were tested (*n* = 5 per group, Table 1): standard configuration (one medial–lateral (ML) and one anterior–posterior (AP) screw); blocked configuration (identical to the standard group but with additional radial grub screws inserted perpendicular to the locking screws in order to eliminate screw–nail clearance and create a mechanically constrained quasi-fixed-angle condition; no integrated threaded locking interface or internal inlay was present); free-hand configuration (with the AP screw inclined by 4°); two medial–lateral screws with 10 mm interscrew distance; two medial–lateral screws with 34 mm interscrew distance; and a three-screw configuration consisting of two medial–lateral and one anterior–posterior screw. Drill holes within the bone substitute were created using a precision milling machine (FP 2, Friedrich Deckel AG, Munich, Germany) with a circular dividing table to ensure angular accuracy (± 0.1°) and interscrew distance precision (± 0.05 mm) for reproducibility. Screw insertion was likewise performed using the same milling machine setup to maintain controlled alignment and positioning.

**Table 1:** Overview of the tested distal locking configurations and screw geometries. ML denotes mediolateral screw orientation, and AP denotes anteroposterior screw orientation. Screw positions are given as longitudinal distances (in mm) measured along the nail axis from the distal nail end (nail tip) to the respective screw axis

Configuration	Screw 1			Screw 2			Screw 3		
	position	direction	angle	position	direction	angle	position	direction	angle
Standard	5 mm	ML	0°	15 mm	AP	0°	-	-	-
Blocked	5 mm	ML	0°	15 mm	AP	0°	-	-	-
Free hand	5 mm	ML	0°	15 mm	AP	4°	-	-	-
Distance 10 mm	5 mm	ML	0°	15 mm	ML	0°	-	-	-
Distance 34 mm	5 mm	ML	0°	39 mm	ML	0°	-	-	-
Three Screws	5 mm	ML	0°	15 mm	AP	0°	25 mm	ML	0°

### Experimental setup

**Fig. 2:**
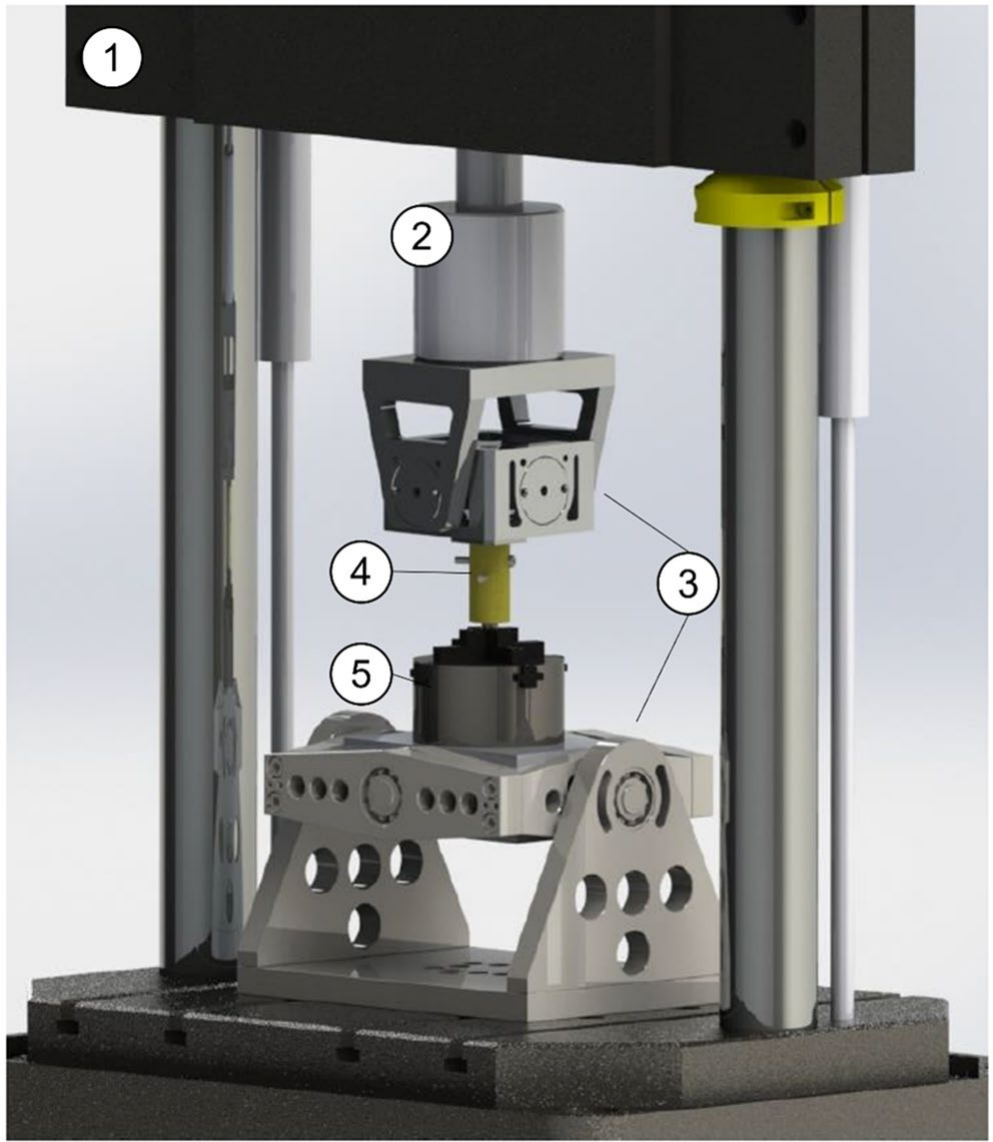
Test setup (1: frame of servo-hydraulic testing machine, 2: actuator with load cell, 3: cardan joints, 4: specimen, 5: jaw chuck)

Cyclic loading was applied using a servo-hydraulic testing system (Instron 8874, Darmstadt, Germany) equipped with a biaxial load cell (± 10 kN, ± 100 Nm, Fig. 2). The specimens were mounted coaxially between two universal joints to avoid undesired bending moments and secondary stresses. The nail dummy was fixed proximally via its square section in a precision jaw chuck, while the embedded synthetic bone cylinder was clamped on the piston in a corresponding fixture aligned with the actuator axis.

A combined axial–torsional loading protocol was applied to simulate physiological conditions acting on intramedullary implants [24]. Axial compression was introduced in a sinusoidal waveform, starting at a load range between 50 N and 250 N. The upper load limit was increased stepwise by 25 N every 2000 cycles, corresponding to a gradual rise in functional loading typically observed during postoperative weight bearing. The initial maximum of 250 N reflects the approximate partial weight bearing load of 25 kg that patients are allowed to apply to the operated limb in early rehabilitation phase [25]. Each 1000 cycles, the test paused at a constant preload of 50 N to record the relative displacement and rotation of the nail within the synthetic bone.

Superimposed on the axial loading, a constant-amplitude torsional moment of ± 2 Nm was applied in synchrony with the axial load, both reaching their maxima simultaneously at a frequency of 2 Hz. This frequency approximates the stride frequency of human gait, which ranges between 1.5 and 2 Hz during normal walking [26], thereby representing physiological cyclic loading conditions encountered during daily activities such as walking or stair climbing [24]. The torsional moment was kept constant throughout the test rather than progressively increased to isolate the effects of rising axial load on the screw–bone interface. Further, confounding influences of simultaneous torque escalation should be prevented.

Testing continued until construct failure, defined as either fracture of the synthetic bone material or loss of fixation stability. The primary outcome measure were the number of cycles to 1 mm relative axial displacement and 0.5° angular rotation between the nail dummy and bone model, which served as indicators of interface motion. Relative axial displacement and torsional rotation were recorded at predefined intervals during cyclic loading, with measurements performed every 1000 cycles during a temporary load stop at a constant axial preload of 50 N. Furthermore, load and cycles to failure were investigated and modes of failure were photo documented.

### Statistical analysis

Statistical analysis was performed using IBM SPSS Statistics 19 (New York, USA). To assess the endurance behavior of the different locking configurations under cyclic loading, a non-parametric survival analysis was conducted using the Kaplan–Meier estimator. This approach allowed the estimation of survival functions describing the probability of construct integrity over the number of load cycles.

Group differences were analyzed using the Log-Rank test for pairwise comparisons. The level of statistical significance was set at α = 0.05. Mean values and standard deviations were additionally calculated for the parameters cycles to failure, cycles to 1 mm relative movement, cycles to 0.5° rotation, and load to failure.

Specimens of the 10 mm screw distance group were excluded from the Kaplan–Meier analysis for the endpoints “1 mm displacement” and “0.5° rotation,” as these samples failed before reaching either criterion. Their premature failure would otherwise have biased the estimation of survival probabilities across groups. Accordingly, the Kaplan–Meier curves were generated for the remaining five configurations, providing a robust comparison of migration resistance and interface stability under combined axial and torsional loading.

## Results

### Construct endurance

**Fig. 3:**
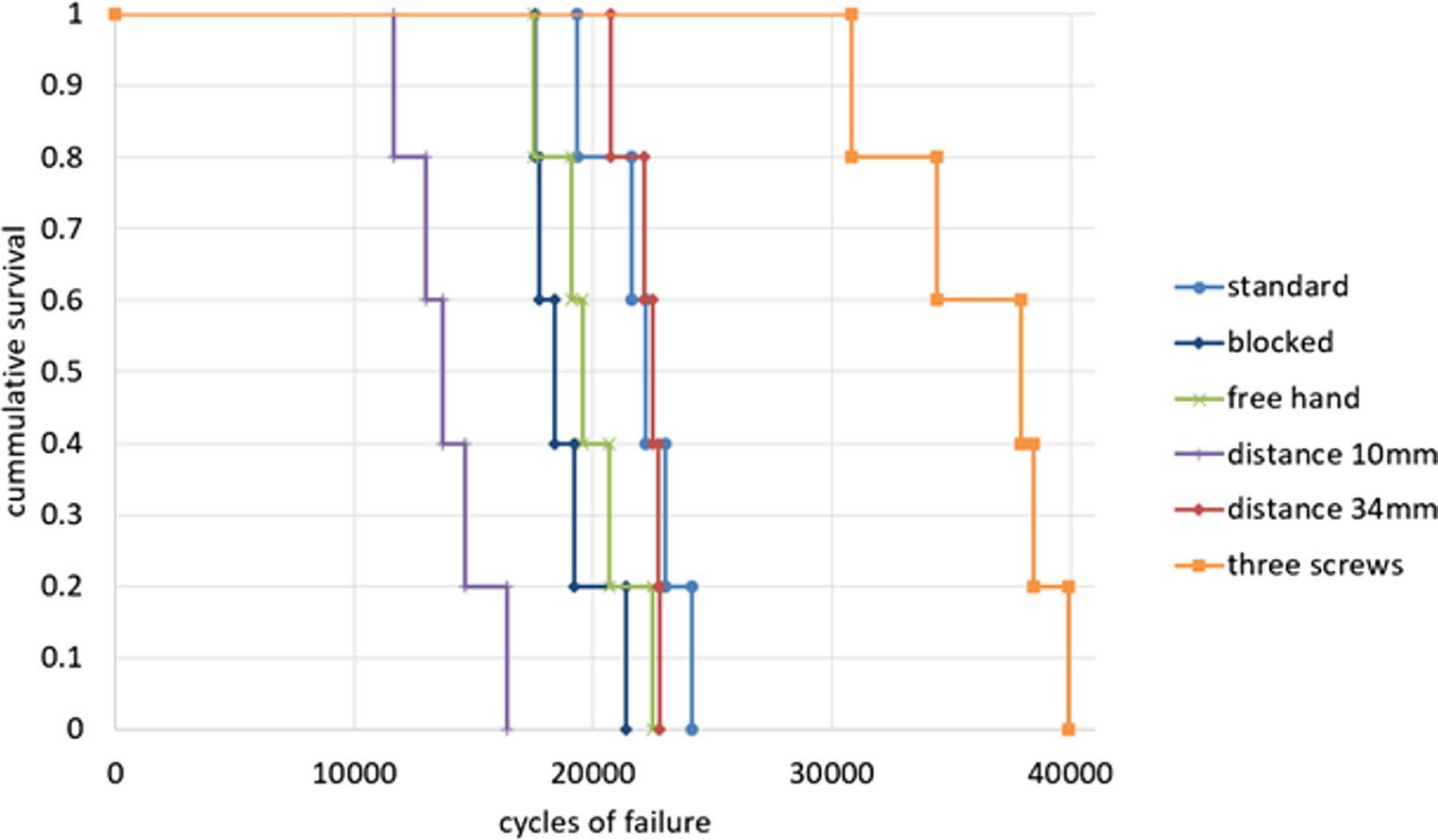
Survival functions (Kaplan-Meier) for cycles to failure (light blue: standard configuration with one ML and one AP screw; dark blue: blocked configuration identical to standard group but with set screws eliminating clearance between nail and locking screws; green: free-hand locking with the AP screw intentionally inclined 4° to mimic free hand locking; purple: two medial–lateral screws spaced 10 mm apart; red: two medial–lateral screws spaced 34 mm apart; and orange: a three-screw configuration with two medial–lateral and one anterior–posterior screw)

All specimens failed by progressive cut-through of the locking screws within the synthetic bone material, resulting in loss of fixation. The number of cycles to failure differed markedly among configurations (Fig. 3). The three-screw configuration exhibited the highest migration resistance, reaching 36,290 ± 3,277 cycles to failure, which was significantly higher than the standard configuration (*p* = 0.002, Log-Rank test).

The standard and 34 mm distance configurations achieved comparable endurance (22,074 ± 1,600 and 22,187 ± 745 cycles, respectively; *p* > 0.05). The blocked and free-hand configurations failed earlier, after 18,859 ± 1,403 and 19,869 ± 1,671 cycles, but these differences were not statistically significant compared with the standard configuration (*p* > 0.05). The 10 mm distance configuration showed the lowest endurance, failing after 13,871 ± 1,587 cycles, which was significantly lower than the standard configuration (*p* = 0.002).

The maximum load sustained before failure mirrored these findings. The three-screw configuration reached the highest load at failure (690 ± 37 N), followed by the standard and 34 mm spacing groups (both 520 ± 24 N and 520 ± 10 N, respectively). Lower loads were recorded for the blocked (470 ± 19 N), free-hand (485 ± 25 N), and 10 mm distance (410 ± 25 N) configurations.

Kaplan–Meier survival analysis confirmed these results. The three-screw configuration demonstrated the longest survival probability across the tested range compared with all other groups (*p* = 0.002). In contrast, the 10 mm spacing group exhibited the steepest decline in survival and the shortest lifetime (*p* = 0.002 vs. standard). The 34 mm and standard configurations showed a trend toward higher survivability compared with the blocked configuration (+ 15%), although this difference did not reach statistical significance (*p* = 0.06).

### Interface motion

**Fig. 4:**
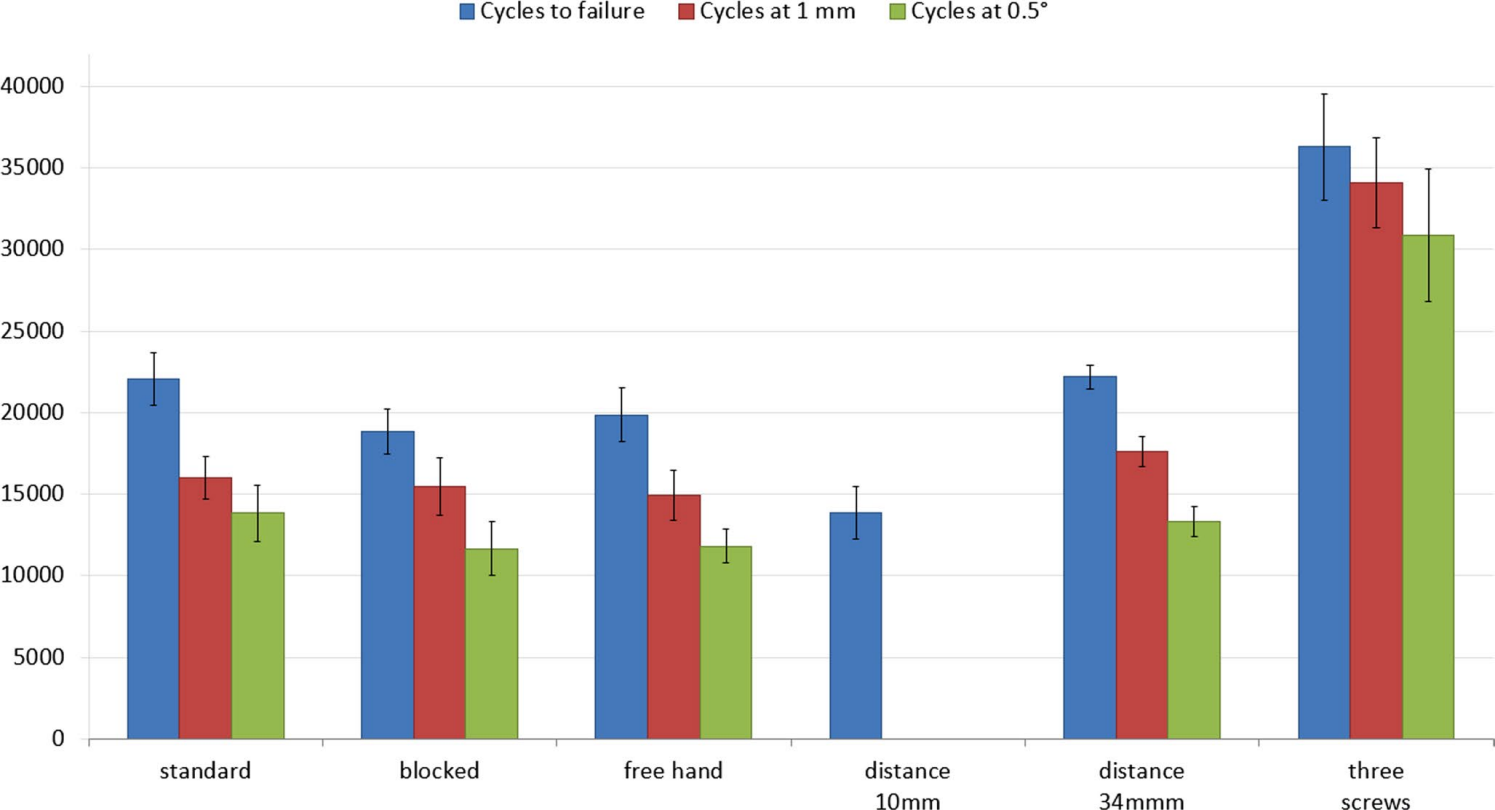
Cycles to failure and cycles to predefined migration thresholds for the different distal locking configurations. Bars represent mean cycles to failure (blue), cycles to 1 mm axial displacement (red), and cycles to 0.5° rotational displacement (green). Error bars indicate standard deviations. The 10 mm distance configuration failed before reaching the migration thresholds and was excluded from those analyses

Relative motion at the nail–bone interface was quantified using three outcome measures: cycles to failure, cycles to 1 mm axial displacement, and cycles to 0.5° rotational movement Fig. 4.

For axial migration, the standard, blocked, and free-hand configurations reached 1 mm relative displacement after approximately 15,000–16,000 cycles. Increasing the interscrew distance to 34 mm delayed this event to 17,620 ± 903 cycles. In contrast, the three-screw configuration required 34,089 ± 2,756 cycles to reach the same threshold, which was significantly higher than the standard configuration (*p* = 0.002). The 10 mm distance configuration failed before reaching 1 mm displacement and was therefore excluded from the analysis for this parameter.

For rotational migration, the three-screw configuration again demonstrated the highest resistance, reaching 0.5° rotation after 30,869 ± 4,055 cycles, significantly later than the standard configuration (*p* = 0.002). The standard and 34 mm distance configurations reached 0.5° rotation after 13,834 ± 1,694 and 13,353 ± 933 cycles, respectively, without a significant difference between them (*p* > 0.05). The blocked and free-hand configurations reached this threshold earlier (11,677 ± 1,625 and 11,834 ± 1,029 cycles, respectively), also without significant differences compared with the standard configuration. As observed for axial displacement, the 10 mm distance configuration failed before reaching 0.5° rotation and was excluded from the analysis.

Overall, the three-screw configuration demonstrated the highest resistance to both axial and rotational migration as well as the highest cycles to failure, whereas reduced screw spacing (10 mm) markedly compromised stability under cyclic loading.

### Failure patterns

**Fig. 5:**
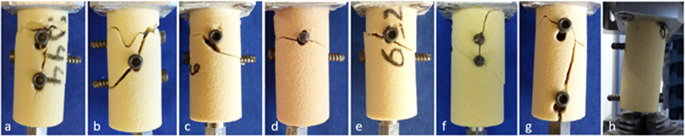
Common failure modes; (**a**) three screws (medial), (**b**) three screws (anterior), (**c**) standard configuration, (**d**) blocked, (**e**) freehand, (**f**) distance 10 mm, (**g**) distance 34 mm, (**h**) specimen tilting representatively shown for configuration with distance 34 mm (anterior)

Visual inspection of the specimens revealed consistent fracture morphologies within and across configurations (Fig. 5). In constructs stabilized with two screws, failure typically initiated as an oblique torsional fracture propagating from the distal screw hole toward the proximal one. This fracture pattern was observed in the standard, blocked, and free-hand configurations and coincided with the onset of rapid increases in displacement and rotation during late cyclic loading.

The 10 mm distance configuration exhibited an additional vertical fracture line between both screws, indicating localized stress concentration in the narrow interscrew region. This group also showed the earliest onset of material cracking and the shortest predicted lifetime, confirming that reduced spacing leads to higher stress accumulation and early interface failure.

In contrast, the 34 mm spacing configuration developed a similar vertical crack between the two screws but only at higher load levels and after substantially more cycles. The increased distance between screws appeared to distribute bending and torsional stresses more evenly along the synthetic bone segment, delaying fracture propagation.

The three-screw configuration displayed a more complex, multiplanar fracture pattern. In medial view, an almost vertical crack formed between the two medial–lateral screws, while in anterior view, an oblique fracture extended through the anterior–posterior screw toward the heads of the adjacent medial–lateral screws. In several specimens, an additional horizontal fracture developed below the most distal screw. These intersecting planes of failure suggest that load transfer occurred across multiple directions, reducing localized stress and contributing to the superior endurance observed for this configuration.

At advanced load stages, several specimens exhibited construct tilting (Fig. 5h), leading to asymmetric cut-through of either the screw heads or tips relative to the synthetic bone surface. The direction of tilting varied randomly and was independent of locking mode or screw orientation. Such asymmetric cut-through is typical once cyclic deformation exceeds the elastic range of the screw–bone interface and reflects local yielding of the bone substitute rather than systematic differences between groups.

## Discussion

The objective of this biomechanical study was to systematically investigate how different distal locking screw configurations of intramedullary nails influence the mechanical stability of the screw–bone interface under combined axial and torsional cyclic loading. Using a standardized synthetic bone model, the results clearly demonstrated that both the number of locking screws and their spatial configuration substantially affect construct endurance and interface motion. Among all tested configurations, the three-screw configuration - featuring multiplanar fixation with two medial–lateral and one anterior–posterior screw - provided the highest migration resistance, greatest load to failure, and lowest micromotion at the screw-bone interface. This finding supports the hypothesis that multiplanar fixation enhances mechanical stability of distal nail constructs. Conversely, the configuration with a short 10 mm interscrew distance produced the earliest failure, reflecting stress concentration occurring between closely spaced screws.

Angular-stable fixation concepts are known to reduce screw toggle by minimizing clearance at the screw–implant interface, particularly in locking plate systems [8, 9]. Translating fixed-angle principles to intramedullary nails has been associated with increased construct stiffness and reduced interfragmentary motion under selected loading conditions [13, 14]. However, fatigue-oriented investigations have shown that such stiffness gains do not necessarily translate into proportional improvements in endurance, particularly when stress concentrations arise at the locking interfaces [15].

In the present study, eliminating screw–nail clearance alone did not improve cut-through performance at the screw–bone interface, as the blocked configuration did not outperform the standard configuration. Instead, migration resistance was primarily governed by screw number and spatial distribution, with multiplanar fixation and increased interscrew distance providing the greatest benefits. This observation is fully consistent with earlier quasi-static investigations from our group, which demonstrated that angular-stable locking effectively reduces clearance but has only minor and inconsistent effects on construct stiffness compared with variations in screw number and interscrew distance [21].

The superior performance of the three-screw configuration is likely attributable to improved multidirectional load distribution and a larger effective moment arm. By distributing bending and torsional loads across orthogonal screw planes, local stress concentrations at the screw–bone interface were reduced. This observation is consistent with previous biomechanical studies identifying screw number and spatial distribution as key determinants of intramedullary nail stability [10, 11, 13].

In contrast, reducing the interscrew distance to 10 mm markedly accelerated construct failure. The fracture morphology of this group showed an additional vertical crack between the screws, confirming localized stress accumulation in the narrow region of overlap. Such behavior has been described previously for densely spaced screws or plates, where reduced lever arms increase bending moments and concentrate cyclic stress in the intervening material [27]. From a mechanical point of view, the reduced spacing diminishes the construct’s ability to distribute load evenly, promoting early yielding of the bone substitute and loss of fixation.

Blocked and free-hand configurations demonstrated comparable behavior, supporting earlier findings from our group that reduction of screw–nail clearance alone does not consistently improve overall construct performance compared with variations in screw number and interscrew distance [21]. Additionally, the blocked configuration required supplementary drill holes for insertion of the radial grub screws, which may have locally weakened the surrounding synthetic material and thereby partially counteracted potential biomechanical advantages of toggle elimination.

The fracture patterns observed in this study provide further insight into the underlying mechanics. Two-screw configurations predominantly failed through oblique torsional cracks along the screw holes, while the three-screw setup produced more complex, intersecting fracture planes, reflecting a more homogeneous stress distribution. This observation directly supports earlier findings by Hoffmann et al. [21], who identified screw number and interscrew distance as the dominant determinants of mechanical performance in intramedullary nailing, exceeding the influence of angular stability alone. The gradual cut-through of screw threads within the polyurethane model mirrors the clinical phenomenon of screw migration or loosening seen in osteoporotic metaphyseal fractures [1, 28], reinforcing the relevance of these findings for implant design and surgical technique.

Several limitations must be acknowledged. First, a synthetic bone substitute was used instead of cadaveric specimens. Although this approach ensured high reproducibility and controlled material properties, it does not fully replicate the anisotropy, viscoelasticity, and heterogeneous structure of human bone [29, 30]. Nevertheless, polyurethane foam models are widely accepted for comparative mechanical testing of osteosynthesis devices because they minimize biological variability and allow reliable interpretation of relative differences between configurations [31, 32]. In addition, the polyurethane bone model did not include a cortical shell or defined fracture morphology and therefore represented a simplified homogeneous cancellous surrogate without physiological cortical load sharing. Furthermore, the intentionally oversized 17 mm canal diameter minimized direct nail–bone contact in order to isolate the mechanical contribution of the distal locking configuration to screw–bone interface stability. In vivo, particularly after reaming, additional load transfer through cortical contact within the isthmus contributes to construct stability and may reduce screw loading. Consequently, the present setup represents a simplified worst-case metaphyseal fixation scenario, and direct clinical transferability should therefore be interpreted with caution. While this simplification does not fully reflect the structural heterogeneity of human metaphyseal bone, it deliberately accentuates screw–bone interface loading and can be considered a worst-case scenario for distal metaphyseal or osteoporotic fracture fixation, allowing robust comparison of different locking configurations. Second, tilting of the constructs during advanced loading stages produced asymmetric cut-through on one screw side, precluding precise quantification of individual screw tip migration. However, this tilting occurred randomly across all groups and did not systematically influence the comparative outcomes. Third, the torsional moment was kept constant at ± 2 Nm rather than scaled with increasing axial load. This choice was deliberate to isolate the effect of axial load increments on the screw–bone interface and follows established protocols for cyclic testing under combined loading. Despite these limitations, the strong agreement across all measured parameters—cycles to failure, displacement, rotation, and load at failure—supports the robustness of the observed trends. The consistent ranking of configurations and the convergence of survival analysis results confirm the internal validity of the data.

The present findings highlight that locking configuration is a key determinant of intramedullary nail fixation strength, not only at the construct level but also at the bone–screw interface, which is the critical zone for long-term stability [22]. In distal or metaphyseal fractures, where cortical support is limited and screw purchase is reduced, applying multiplanar fixation with sufficient interscrew distance can substantially enhance migration resistance and delay failure. From a biomechanical perspective, the present findings suggest potential advantages of three-screw distal locking patterns—combining medial–lateral and anterior–posterior screws—whenever anatomical space allows. Conversely, closely spaced screws should be avoided in metaphyseal segments, as they promote early cut-through and construct loosening, particularly in osteoporotic bone.

From a design perspective, the findings reinforce ongoing trends toward angle-stable interlocking mechanisms and multiplanar distal locking options in modern intramedullary nails. This perspective directly builds on earlier quasi-static investigations from our group, which demonstrated that optimal mechanical performance of intramedullary nailing is achieved by combining angular stability to reduce screw–nail clearance with an optimized arrangement of locking screws to increase construct stiffness [21]. Such configurations may improve postoperative stability, allow earlier weight bearing, and reduce the risk of secondary loss of reduction in clinical practice.

## Conclusion

Concluding, distal locking configuration had a decisive influence on screw–bone interface stability under combined axial–torsional cyclic loading. The three-screw multiplanar configuration achieved the highest construct endurance, greatest load to failure, and lowest micromotion, confirming that multiplanar fixation enhances stress distribution and delays failure. In contrast, a 10 mm interscrew distance markedly reduced construct endurance due to stress concentration between adjacent screws. Standard and 34 mm spacing performed comparably, while blocked and free-hand variants offered no additional benefit in terms of construct endurance. Overall, the configuration with three multiplanar locking screws and sufficient screw separation demonstrated the highest interface stability within the limitations of the present experimental model, especially in metaphyseal regions with limited cortical support, and may contribute to improved construct durability under cyclic loading conditions.

## Data Availability

The datasets generated and/or analyzed during the current study are available from the corresponding author on reasonable request.
